# Alterations in gene expression induced by cyclic mechanical stress in trabecular meshwork cells

**Published:** 2009-03-11

**Authors:** Coralia Luna, Guorong Li, Paloma B. Liton, David L. Epstein, Pedro Gonzalez

**Affiliations:** Department of Ophthalmology, Duke University, Durham, NC

## Abstract

**Purpose:**

To investigate the changes in gene expression induced by cyclic mechanical stress (CMS) in trabecular meshwork (TM) cells.

**Methods:**

Human TM cultures from three donors were plated on type I collagen-coated flexible silicone bottom plates and subjected to 15% stretching, one cycle per second for 6 h. Non-stressed parallel control cultures were incubated under the same conditions in the absence of CMS. Total RNA from each culture was amplified (1 round of amplification) and hybridized to Operon Human Oligo Arrays version 3.0 (35 K probes). Differences in gene expression induced by CMS were analyzed using Genespring 7.2. quantitative polymerase chain reaction (Q-PCR) was used to confirm changes in the expressions of 12 selected genes. The effects of chemical inhibitors for p38, ERK (extracellular signal-regulated kinase), JNK (Jun N-terminal kinase), PKA (protein kinase A), PI3K (phosphoinositide 3-kinase), and P2 (purinergic 2) receptors on the induction of MMP3 (matrix metalloproteinase 3), HSP70 (heat shock protein 70), ECSM1 (endothelial cell specific molecule 1), BMP2 (bone morphogenetic protein 2), VEGFC (vascular endothelial growth factor C), and IL-8 (interleukin 8) were evaluated in porcine TM cells subjected to the same regime of CMS as that used in human cells.

**Results:**

CMS induced extensive gene expression changes (664 genes, p≤0.05) twofold or higher in cultured TM cells. Many of these changes were related to extracellular matrix (ECM) synthesis and remodeling including the upregulation of two metalloproteinases (*MMP3* and *MMP10*). Cytoskeleton and cell adhesion genes were also affected by CMS as well as genes known to be involved in cellular protection against stress including several members of the HSP70 family. Inhibition of PI3K/AKT and P2 receptors pathways significantly reduced the induction of *MMP3* and *IL-8* whereas the inhibition of the PKA/cAMP pathway decreased *ECSM1* and *BMP2*.

**Conclusions:**

CMS activated many genes that could influence the aqueous humor outflow facility, specifically genes involved in ECM synthesis and remodeling (e.g. MMPs), cytoskeletal organization, and cell adhesion. Induction of *MMP3* has the potential to increase the aqueous humor outflow facility and could be part of a homeostatic mechanism involved in the maintenance of normal intraocular pressure (IOP) levels. Other observed changes are more likely to be related to general cellular responses to stress (e.g., *HSP70*, *ECSM1*, and *BMP2*). Although these latter changes may initially help to repair mechanical damage, some of them such as the induction of *BMP2* could eventually increase tissue rigidity and compromise the ability of the TM to maintain normal levels of outflow resistance.

## Introduction

The trabecular meshwork (TM) and Schlemm’s canal form the major conventional route for aqueous outflow from the anterior chamber of the human eye. The TM is also the site of the abnormal increase in outflow resistance that leads to elevated intraocular pressure (IOP) in glaucoma [1-4]. Similar to other tissues in the body, the TM is subjected to mechanical forces that can exert important effects on the normal physiology of the tissue as well as contribute to pathological alterations [5,6].

Several studies have demonstrated that the TM responds to the stretch produced by a static increase in IOP by altering both its morphology and patterns of gene expression [7-10]. Such changes in gene expression have been proposed to play a role in restoring normal levels of IOP through homeostatic influences on aqueous humor outflow facility [7,9].

However, mechanical stress in the TM in vivo does not only result from simple static changes in IOP. In vivo, the TM is constantly subjected to transient spikes of IOP such as those associated with systole of the cardiac cycle, blinking, and eye movement [1,11]. In particular, the cardiac cycle leads to oscillations of IOP potentially in the order of 2.7 mmHg, which then produces cycles of TM tissue stretching and relaxing [11].

In several cell types, cyclic regimes of mechanical stress are known to exert different effects from static stretching [5]. Therefore, it should be expected that cyclic mechanical stimulation of TM cells might elicit different responses from those observed after static stretching.

Surprisingly, Ramos and Stamer [12] recently reported that cyclic IOP in perfused anterior segments of human and porcine eyes resulted in a significant decrease in outflow facility. These changes in outflow facility were not associated with detectable damage to the cells or structures of the outflow pathway, suggesting that it may result from active cellular responses to the cyclic mechanical stimulus.

A better characterization of the cellular responses to cyclic mechanical stress (CMS) in the TM is needed to understand the influences of the biomechanical environment on the physiologic function of the conventional outflow pathway.

To gain insight into these mechanisms, we investigated the changes in gene expression induced by cyclic mechanical stress in cultured TM cells using gene microarrays. We also analyzed the potential involvement of several regulatory pathways on these observed changes in gene expression.

## Methods

### Cell cultures

Within 48 h post mortem, human trabecular meshwork (HTM) cell cultures were obtained from cadaver eyes that did not have any history of eye disease [13]. Tissues were manipulated in accordance with the Declaration of Helsinki. Three HTM primary cell lines (from ages 14, 16, and 25 year old eyes) were used in these experiments. Porcine TM (pTM) cells were generated from fresh pig eyes using the same protocol. Cell cultures were maintained at 37 °C in 5% CO_2_ in media (low glucose Dulbecco’s Modified Eagle Medium [DMEM] with L-glutamine, 110 mg/ml sodium pyruvate, 10% fetal bovine serum, 100 µM non-essential amino acids, 100 units/ml penicillin, 100 µg/ml streptomycin sulfate, and 0.25 µg/ml amphotericin B). All the reagents were obtained from Invitrogen Corporation (Carlsbad, CA).

### RNA isolation and quantitative polymerase chain reaction

HTM and pTM primary cell cultures were washed with phosphate buffered saline (PBS) and immediately submerged in RNA-later (Ambion Inc., Austin, TX). Total RNA was isolated using an RNeasy kit (Qiagen Inc., Valencia, CA) according to the manufacturer’s instructions and then treated with DNase. RNA yields were measured using RiboGreen fluorescent dye (Molecular Probes, Eugene, OR). First strand cDNA was synthesized from total RNA (1 µg) by reverse transcription using oligodT and Superscript II reverse transcriptase (Invitrogen Corporation) according to the manufacturer’s instructions. Quantitative polymerase chain reactions (Q-PCR) were performed in a 20 µl mixture that contained 1 µl of the cDNA preparation and 1X iQ SYBR Green Supermix (Bio-Rad, Hercules, CA), using the following PCR parameters: 95 °C for 5 min followed by 50 cycles of 95 °C for 15 s, 65 °C for 15 s, and 72 °C for 15 s. The fluorescence threshold value (C_t_) was calculated using the iCycle system software (Bio-Rad, Hercules, CA). The absence of nonspecific products was confirmed by both the analysis of the melt curves and by electrophoresis in 3% Super AcrylAgarose gels. β-Actin (*ACTB*) was used as an internal standard of mRNA expression. This gene was selected as a control because it did not show any significant difference in expression in the array analysis. The primers used for Q-PCR amplification are shown in Table 1.

**Table 1:** Primers used for Q-PCR amplification.

**Gene**	**Forward**	**Reverse**
**Human genes**		
β-Actin	5′-CCTCGCCTTTGCCGATCCG-3′	5′-GCCGGAGCCGTTGTCGACG-3′
*HSP70B'*	5′-ACAGGAGCACAGGTAAGGCT-3′	5′-TTCATGAACCATCCTCTCCA-3′
*MMP10*	5′-TGCATCAGGCACCAATTTAT-3′	5′-TGTTGGCTGAGTGAAAGAGC-3′
*ECSM1*	5′-TTTCTCTCACGGAGCATGAC-3′	5′-GGCAGCATTCTCTTTCACAA-3′
*MMP3*	5′-GCCAGGGATTAATGGAGATG-3′	5′-ATTTCATGAGCAGCAACGAG-3′
Regulator of G protein 20	5′-GAAGATCAGAGGCCCACAAT-3′	5′-GGCGTTGACTTCTTCCAGAG-3′
*BMP2*	5′-GGACGCTCTTTCAATGGAC-3′	5′-ACCATGGTCGACCTTTAGGA-3′
Hyaluronan synthase	5′-CACACAGACAGGCTGAGGAC-3′	5′-TCCAAAGAGTGTGGTTCCAA-3′
*VEGFC*	5′-GGATGCTGGAGATGACTCAA-3′	5′-TTCATCCAGCTCCTTGTTTG-3′
JunB proto-oncogene	5′-CGATCTGCACAAGATGAACC-3′	5′-GCTGCTGAGGTTGGTGTAAA-3′
Nuclear receptor subfamily 4A	5′-GAAGCTGAGATGCCCTGTATC-3′	5′-ATGGTGGGCTTGATGAACTC-3′
Basic transcription element	5′-GGCTGTGGGAAAGTCTATGG-3′	5′-CCGTTCACCTGTATGCACTC-3′
Early growth response 3	5′-GCTGAACTGGGCTGTGTTTA-3′	5′-ACATGATTTCAGAGCGGATG-3′
**Porcine genes**		
β-Actin	5′-AAGATCAAGATCATCGCGCCTCCA-3′	5′-TGGAATGCAACTAACAGTCCGCCT-3′
*HSP70*	5′-CCACCAAGGATGCGGGGGTA-3′	5′-GCGCTCCCCCTTGCCCGTCC-3′
*MMP3*	5′-TTTTGCAGTTCGAGAACACG-3′	5′-TGAAAGAGACCCAGGGAATG-3′
*ECSM1*	5′-TGTTACCGCACAGTCTCAGG-3′	5′-TGACAGCTGCAAGTCTGCTT-3′
*BMP2*	5′-ACCAACCTGGTGTCCAAAAG-3′	5′-GTCCCCACCGAGGAGTTTAT-3′
*VEGFC*	5′-TTCCTCTAATGCCGGAGATG-3′	5′-CACACGAGTTGGGGAAAAGT-3′
*IL8*	5′-AAACTGGCTGTTGCCTTCTT-3′	5′-ATTTATGCACTGGCATCGAA-3′

List of all primers used in this study to validate the microarray data by Q-PCR. *b-Actin* was used as the normalizing control gene for all comparisons.

### Cyclic mechanical stress application in cell culture

HTM (passage 3) and pTM (passage 4) cultures were plated on type I collagen-coated flexible silicone bottom plates (Flexcell, Hillsborough, NC). One day after confluence, culture medium was switched to serum-free DMEM 3 h before cyclic mechanical stress. Cells were stressed for 6 h (15% stretching, 1 cycle/s), using the computer-controlled, vacuum-operated FX-3000 Flexercell Strain Unit (Flexcell). A frequency of 1 cycle/s was selected to mimic cardiac frequency. Since it is difficult to estimate the amount of stress to which TM cells are subjected in vivo but IOP can change as much as 15 mmHg in 10 s [11], we selected 15% stretching because at 1 cycle/s, it was high enough to induce detectable changes in gene expression while exerting only minimal effects on cell survival for both porcine and human TM cells. Control cells were cultured under the same conditions, but no mechanical force was applied.

### Gene microarray and data analysis

Total RNA from three HTM cell cultures stressed or held static were amplified (one round amplification) and hybridized to Operon Human Oligo Arrays version 3.0 (35 K probes; Eurofins Operon, Huntsville, AL) at the Duke University Microarray facility (Durham, NC). The Human Genome Oligo Set Version 3.0 represents 24,650 genes and 37,123 gene transcripts. Raw data was normalized and analyzed using GeneSpring 7.2 (Silicon Genetics, Wilmington, DE). Genes were filtered to their intensities in the control channel (control used was Universal Reference Human RNA from Stratagene, Huntsville, AL). Raw data values below 100 were considered unreliable. Intensity-dependent normalization was performed per spot and per chip (LOWESS, locally weighted scatterplot smoothing). An ANOVA test was performed (p values≤0.05 were considered significant) for genes differentially expressed using the Benjamin and Hochberg False Discovery Rate correction test.

### Cell viability assay

Cell viability was assayed after 6 h of CMS by measuring lactate dehydrogenase released to the culture media as a result of plasma membrane damage. The Cito Tox 96® Non-Radioactive Cytotoxicity assay (Promega, Madison, WI) was used to carry out the measurement following manufacturer’s instructions.

### Inhibitors

Chemical inhibitors for p38 (SB203580), mitogen-activated protein kinase kinase and extracellular regulated kinase (MEK/ERK; PD98059), Janus kinase protein (JNK; SP600125), protein kinase A (PKA; H89), phosphoinositide 3-kinase (PI3K; wortmannin), and P2 receptors (suramin) were all from Sigma (St Louis, MO). The inhibitors were used at a concentration of 10 µM 1 h before and during the stretching.

## Results

### Changes in gene expression after mechanical stress in human trabecular meshwork cells

HTM cells were subjected to mechanical stress for 6 h to evaluate the effects of CMS on the gene expression profile. Gene array analysis showed statistically significant (p≤0.05) 2.0 fold or higher changes in 664 gene transcripts. Out of these 664 genes, 349 were upregulated and 315 were downregulated. The genes most highly upregulated or downregulated after CMS are shown in Table 2.

**Table 2:** Genes whose expression was found to change by greater than five fold after exposure to CMS.

**Genes upregulated and downregulated in HTM cells after CMS**	**Fold**	**p value**	**GenBank accession number**
Heat shock 70 kDa protein 6 (*HSP70B'*)	489.4	0.0206	NM_002155
Protein phosphatase 1, subunit ^14^C	66.58	2.84E-05	NM_030949
Integrin, beta 6	59.16	0.000405	NM_000888
Regulator of G-protein signaling 20	53.81	0.000828	NM_003702
Chromosome 9 open reading frame 26 (*NF-HEV*)	40.98	5.60E-05	NM_033439
Endothelial cell-specific molecule 1	33.41	0.000613	NM_007036
Matrix metalloproteinase 3 (stromelysin 1)	29.78	1.13E-05	NM_002422
Matrix metalloproteinase 10 (stromelysin 2)	26.72	0.000597	NM_002425
Nm23-phosphorylated unknown substrate	23.27	1.77E-05	NM_032873
Bone morphogenetic protein 2	20.07	0.000553	NM_001200
Neuronal protein	19.73	0.0002	NM_013259
Hypothetical protein FLJ23657	17.56	0.000366	AK074237
T-cell activation kelch repeat protein	17.12	0.000116	NM_032505
Parathyroid hormone-like hormone	16.73	0.0055	NM_002820
Hypothetical protein FLJ12604	15.98	0.000264	NM_024621
Kruppel-like factor 7 (ubiquitous)	15.62	0.000214	BC012919
Fibroblast growth factor 1 (acidic)	13.75	0.00479	NM_000800
Hypothetical protein FLJ13391	12.28	5.88E-05	NM_032181
Galanin	10.68	0.000155	NM_015973
ATPase family, AAA domain containing 3B	10.64	1.38E-05	NM_031921
Pentaxin-related gene	9.761	0.00016	NM_002852
Polymerase (RNA) III polypeptide D	9.255	4.25E-06	NM_001722
Metallothionein 1G	8.385	0.0134	BC020757
Hypothetical protein MGC11324	8.315	0.00094	NM_032717
Heat shock 70 kDa protein 1B	8.117	0.000324	NM_005346
Potassium voltage-gated channel, member 4	7.964	6.90E-05	NM_004978
Nucleoside phosphorylase	7.604	7.38E-05	NM_000270
Protein phosphatase 1, subunit 15A	7.104	0.000387	NM_014330
Regulator of G-protein signaling 17	6.969	0.000364	NM_012419
Carbohydrate (chondroitin 4) sulfotransferase 11	6.801	0.00106	NM_018413
ADP-ribosylation factor-like 7	6.475	0.000369	NM_005737
Dickkopf homolog 1 (*Xenopus laevis*)	6.464	0.000163	NM_012242
Solute carrier family 17, member 1	6.442	0.00549	NM_005074
BTB (POZ) domain containing 11	6.193	5.74E-05	NM_152322
Thymic stromal lymphopoietin	6.189	0.00254	NM_033035
Mitogen-activated protein kinase kinase 4	6.099	0.00378	NM_003010
Heat shock 105 kDa/110 kDa protein 1	5.997	0.000789	NM_006644
Chemokine (C-C motif) ligand 7	5.941	0.00053	NM_006273
Vascular endothelial growth factor C	5.917	7.05E-05	NM_005429
GTPase activating Rap/RanGAP domain-like 4	5.888	0.00123	AB028962
Iduronidase, alpha-L-	5.829	7.76E-05	NM_000203
T-box 3 (ulnar mammary syndrome)	5.765	6.16E-05	NM_016569
Hyaluronan synthase 2	5.388	0.00474	NM_005328
Heparan sulfate 3-O-sulfotransferase 1	5.377	0.00618	NM_005114
Bone morphogenetic protein 6	5.079	0.00223	NM_001718
Myosin X	5.073	3.50E-05	AB018342
Integrin, alpha 2	5.06	0.0112	NM_002203
Basic transcription element binding protein 1	-13.88	0.0022	NM_001206
Contactin 3 (plasmacytoma associated)	-13.41	0.00967	AB040929
Nuclear receptor subfamily 4, group A, member 2	-13.28	0.000121	NM_006186
Nuclear receptor subfamily 4, group A, member 1	-12.58	0.000702	NM_173158
Chromosome 20 open reading frame 129	-10.39	0.000593	NM_030919
Distal-less homeo box 2	-9.82	2.50E-05	NM_004405
Neuropeptide Y receptor Y1	-9.366	0.00977	NM_000909
Early growth response 3	-8.314	0.000761	NM_004430
FBJ murine osteosarcoma viral oncogene homolog B	-8.036	0.000207	NM_006732
Chromosome 2 open reading frame 11	-7.839	0.00422	NM_144629
B-cell CLL/lymphoma 3	-7.259	0.00165	NM_005178
Chromosome 6 open reading frame 111	-7.021	0.00265	NM_032870
Tumor necrosis factor superfamily, member 10	-6.621	0.000944	NM_003810
F-box protein 32	-6.561	0.00181	NM_058229
Nuclear receptor subfamily 4, group A, member 3	-6.488	0.000476	NM_173200
Solute carrier family 40, member 1	-6.414	0.000916	NM_014585
Phosphodiesterase 5A, cGMP-specific	-6.261	0.00289	NM_001083
Zinc finger protein 36, C3H type-like 2	-5.684	0.000817	NM_006887
Myeloid/lymphoid or mixed-lineage leukemia	-5.466	0.00544	NM_004529
KIAA1199	-5.371	0.00384	AB033025
TGFB inducible early growth response	-5.246	0.000796	NM_005655

Columns show the gene name, the fold expression change determined by microarray analysis, the calculated p value, where p<0.05 was considered to be statistically significant, and the GeneBank accession number.

CMS induced changes in the expression of genes that are known to be involved in cell protection as well as genes that because of their known function could potentially  influence aqueous humor outflow facility. Specifically, CMS affected the expression of genes involved in cellular stress (Table 3) and extracellular matrix (ECM) synthesis and remodeling (Table 4) as well as genes known to affect cytoskeleton and cell adhesion (Table 4 and Table 5).

**Table 3:** CMS-induced changes in HTM cells stress/defense genes.

**Stress induced genes up and down regulated in HTM cells**	**Fold**	**p value**	**GenBank accession number**
Heat shock 70 kDa protein 6 (HSP70B')	489.4	0.0206	NM_002155
Pentaxin-related gene, rapidly induced by IL-1 beta	9.761	0.00016	NM_002852
Metallothionein 1G	8.385	0.0134	BC020757
Heat shock 70 kDa protein 1B	8.117	0.000324	NM_005346
Protein phosphatase 1, regulatory (inhibitor) subunit 15A	7.104	0.000387	NM_014330
Heat shock 105 kDa/110 kDa protein 1	5.997	0.000789	NM_006644
Heat shock 70 kDa protein 8	4.551	0.00105	NM_006597
Heat shock 70 kDa protein 5 (glucose-regulated protein, 78 kDa)	4.268	0.0309	NM_005347
Serum/glucocorticoid regulated kinase	4.265	0.00277	NM_005627
Phosphoprotein with glycosphingolipid-enriched microdomains	4.097	0.000293	NM_018440
DnaJ (Hsp40) homolog, subfamily A, member 1	3.838	0.00456	NM_001539
DnaJ (Hsp40) homolog, subfamily B, member 1	3.79	0.000307	NM_006145
Metallothionein 1B (functional)	3.286	0.00116	NM_005947
BCL2-associated athanogene 3	3.18	0.00513	NM_004281
Metallothionein 2A	3.164	0.0026	NM_005953
similar to *Escherichia coli* DnaJ, but lacks a J-domain	3.108	0.000794	AF395440
UL16 binding protein 2	3.104	0.0157	NM_025217
Metallothionein 1H	3.086	0.00131	NM_005951
Stress-induced-phosphoprotein 1	3.016	0.000709	NM_006819
Metallothionein 1E (functional)	2.92	0.0407	AF495759
Protein phosphatase 1, regulatory subunit 10	2.789	0.0343	NM_002714
Angiopoietin-like 4	2.753	0.00858	NM_139314
DnaJ (Hsp40) homolog, subfamily B, member 4	2.742	0.0108	NM_007034
Heat shock 90 kDa protein 1, alpha-like 3	2.616	0.00257	M30627
Metallothionein 1A (functional)	2.546	0.00823	BC029475
Heat shock 90 kDa protein 1, alpha	2.446	0.00395	NM_005348
Chaperonin containing TCP1, subunit 6A (zeta 1)	2.33	0.00148	NM_001762
Heat shock 90 kDa protein 1, beta	2.306	1.09E-05	NM_007355
Fas (TNFRSF6)-associated via death domain	2.285	0.0025	NM_003824
Heat shock 70 kDa protein 9B (mortalin-2)	2.248	0.00138	NM_004134
Px19-like protein	2.137	0.00876	NM_013237
AHA1	2.133	0.00863	NM_012111
Nuclear receptor subfamily 4, group A, member 2	-13.28	0.000121	NM_006186
Tumor necrosis factor (ligand) superfamily, member 10	-6.621	0.000944	NM_003810
Angiopoietin-like factor	-4.358	0.0103	NM_021146
synonyms: G10P1, IFI56, IFI-56, IFNAI1, RNM561, GARG-16;	-3.888	0.00272	NM_001548
Hypoxia-inducible protein 2	-3.398	0.00368	NM_013332
Interferon regulatory factor 1	-3.185	0.00155	NM_002198
Chemokine-like receptor 1	-3.145	0.00408	NM_004072
Bradykinin receptor B2	-2.916	0.00242	NM_000623
Histone deacetylase 5	-2.88	0.00044	NM_005474
Calcium modulating ligand	-2.562	4.70E-05	NM_001745
Interferon gamma receptor 2 (interferon gamma transducer 1)	-2.546	0.000401	NM_005534
Zinc finger protein 179	-2.425	0.0108	NM_007148
Heat shock transcription factor 2	-2.42	6.74E-07	NM_004506
Oxidation resistance 1	-2.373	0.0148	BC032710
Nuclear factor, interleukin 3 regulated	-2.166	0.000751	NM_005384
Heat shock 27 kDa protein 2	-2.125	0.0199	NM_001541
Collagen, type IV, alpha 3 binding protein	-2.124	0.00719	NM_005713
Prostaglandin-endoperoxide synthase 2	-2.008	0.0142	NM_000963

Genes functionally relevant to cell stress/defense responses that were significantly up- or down-regulated more than two fold after CMS in TM cells. Columns show the gene name, the fold expression change determined by microarray analysis, the calculated p value, where p<0.05 was considered to be statistically significant, and the GeneBank accession number.

**Table 4:** Extracellular matrix changes in gene expression after CMS in HTM cells.

**Gene**	**Fold**	**p value**	**GenBank accession number**
**ECM genes upregulated after CMS**			
**ECM regulators/modifiers**			
Matrix metalloproteinase 3 (stromelysin 1, progelatinase)	29.78	1.13E-05	NM_002422
Matrix metalloproteinase 10 (stromelysin 2)	26.72	0.000597	NM_002425
Bone morphogenetic protein 2	20.07	0.000553	NM_001200
Fibroblast growth factor 1 (acidic)	13.75	0.00479	NM_000800
Carbohydrate (chondroitin 4) sulfotransferase 11	6.801	0.00106	NM_018413
Vascular endothelial growth factor C	5.917	7.05E-05	NM_005429
Hyaluronan synthase 2	5.388	0.00474	NM_005328
Heparan sulfate (glucosamine) 3-O-sulfotransferase 1	5.377	0.00618	NM_005114
Bone morphogenetic protein 6	5.079	0.00223	NM_001718
Carbohydrate (N-acetylglucosamine-6-O) sulfotransferase 2	4.423	0.000172	NM_004267
Heparan sulfate (glucosamine) 3-O-sulfotransferase 3A1	4.329	1.13E05	NM_006042
Tumor necrosis factor, alpha-induced protein 6	4.082	0.00033	NM_007115
Plasminogen activator, tissue	3.236	0.00284	NM_000931
Tissue factor pathway inhibitor 2	2.74	0.00486	NM_006528
Procollagen C-endopeptidase enhancer 2	2.594	0.00373	NM_013363
A disintegrin-like and metalloprotease with thrombospondin type 1 motif, 1	2.081	0.0285	NM_006988
**Proteoglycans**			
Endothelial cell-specific molecule 1	33.41	0.000613	NM_007036
Syndecan 1	2.802	0.00156	NM_002997
Chondroitin sulfate proteoglycan 4 (melanoma-associated)	4.376	0.000852	NM_001897
**ECM genes downregulated after CMS**			
**ECM regulators/modifiers**			
Transforming growth factor, beta receptor III (betaglycan, 300 kDa)	-2.864	0.00214	NM_003243
Thrombospondin 1	-2.883	0.0163	NM_003246
A disintegrin-like and metalloprotease (reprolysin type) with thrombospondin type 1 motif, 5 (aggrecanase-2)	-2.771	0.0196	NM_007038
Transforming growth factor, beta 3	-2.64	0.00823	NM_003239
Bone morphogenetic protein receptor, type IA	-2.494	0.0243	NM_004329
Sulfatase 2	-2.336	0.00141	AB033073
Sulfatase 1	-2.283	0.0124	AB029000
Lysyl oxidase-like 4	-2.144	0.000569	NM_032211
**Proteoglycans**			
Glypican 4	-3.08	0.00368	NM_001448
Osteoglycin (osteoinductive factor, mimecan)	-2.297	0.0105	NM_033014
**Matrix proteins**			
Collagen, type VIII, alpha 1	-2.594	0.0103	NM_001850
Laminin, beta 1	-2.443	0.0023	NM_002291
Collagen, type IV, alpha 3 (Goodpasture antigen) binding protein	-2.124	0.00719	NM_005713

Genes associated with ECM that were significantly up- or down-regulated more than two fold after CMS in TM cells. Columns show the gene name, the fold expression change determined by microarray analysis, the calculated p value, where p<0.05 was considered to be statistically significant, and the GeneBank accession number.

**Table 5:** CMS-induced changes in the expression of cytoskeleton-related genes.

**Cytoskeleton genes upregulated or downregulated in HTM after CMS**			
**Gene**	**Fold**	**p value**	**GenBank accession number**
Ectodermal-neural cortex (with BTB-like domain)	4.998	0.0015	NM_003633
Tubulin, beta, 4	4.489	0.00178	NM_006086
Myosin IXB	3.572	0.000919	NM_004145
Actin related protein 2/3 complex, subunit 5-like	3.519	4.30E-05	NM_030978
Leucine rich repeat (in FLII) interacting protein 1	3.016	0.00186	NM_004735
Syndecan 1	2.802	0.00156	NM_002997
Chromosome 14 open reading frame 31	2.672	0.00977	NM_152330
Kinesin family member 21A	2.588	0.000145	AK000059
Tubulin, beta polypeptide	2.388	0.00293	NM_001069
Leucine rich repeat (in FLII) interacting protein 1	2.212	0.00923	NM_004735
Filamin C, gamma (actin binding protein 280)	2.205	0.00172	NM_001458
Paxillin	2.168	0.00754	NM_002859
Molecule interacting with Rab13	2.153	0.00962	AB051455
Tropomyosin 3	2.126	0.0175	AK092712
Tubulin beta MGC4083	2.119	0.00802	NM_032525
Tubulin alpha 6	2.073	0.000692	NM_032704
Arg/Abl-interacting protein ArgBP2	-4.37	0.000424	NM_021069
Ankyrin repeat, family A (RFXANK-like), 2	-3.24	0.0035	NM_023039
Protein kinase C and casein kinase substrate in neurons 3	-2.913	0.00147	NM_016223
A kinase (PRKA) anchor protein (yotiao) 9	-2.604	0.00434	NM_147185
Syntrophin, beta 2	-2.415	0.00777	NM_006750
Profilin 2	-2.058	0.00371	NM_002628
Downregulated in ovarian cancer 1	-2.036	0.0402	NM_014890

Cytoskeleton related genes that were significantly up- or down-regulated more than two fold after CMS in TM cells. Columns show the gene name, the fold expression change determined by microarray analysis, the calculated p value, where p<0.05 was considered to be statistically significant, and the GeneBank accession number.

Numerous stress defense and cell defense genes showed changes in expression. This group included 14 heat shock and heat shock related genes (Table 3). Heat shock protein 70 B (*HSP70B'*) showed the largest upregulation, and other members of the HSP70 family also showed high levels of induction. In addition, several metallothioneins (1A, 1B, 1E, 1G, 1H, and 2A), angiopoietin-like 4, and cluster of differentiation (CD) antigens were among the upregulated genes.

Several genes coding for proteins that can affect the ECM exhibited expression changes after CMS. Among these, the upregulation of two metalloproteinases, *MMP3* and *MMP10*, with levels of induction of 29 and 26 fold, respectively, was particularly noticeable. Various proteoglycan genes showed either downregulation or upregulation after CMS. The most upregulated was the endothelial cell specific molecule-1 (*ECSM1* or endocan). Genes associated with proteoglycan synthesis and degradation such as hyaluronan synthase and several sulfotransferases were also upregulated. Important ECM structural components such as collagens and laminin showed a decrease in expression. Other significant changes related to the ECM included the upregulation of growth factors (fibroblast growth factor [*FGF*] acidic and vascular endothelial growth factor C**[*VEGFC*]), bone morphogenetic proteins (2 and 6), and plasminogen activator tissue (Table 4).

CMS altered the expression of many cytoskeleton and cell adhesion genes. The most upregulated cytoskeleton gene was an actin binding protein that is also known to be induced by oxidative stress (ectodermal-neural cortex). The most downregulated gene was Arg/Abl-interacting protein (*ArgBP2*), which belongs to a family that regulates both cell adhesion and cytoskeletal organization (Table 5). Among the genes involved in cell adhesion, particularly evident was the increase in expression of integrin beta 6 (Table 6).

**Table 6:** CMS-induced changes in cell adhesion genes.

**Cell adhesion genes upregulated and downregulated in HTM after CMS**			
**Gene**	**Fold**	**p value**	**GenBank accession number**
Integrin, beta 6	59.16	0.000405	NM_000888
Integrin, alpha 2 (CD49B, alpha 2 subunit of VLA-2 receptor)	5.06	0.0112	NM_002203
Chondroitin sulfate proteoglycan 4 (melanoma-associated)	4.376	0.000852	NM_001897
Tumor necrosis factor, alpha-induced protein 6	4.082	0.00033	NM_007115
Leupaxin	3.449	0.000212	NM_004811
Bystin-like	2.625	0.000981	NM_004053
Integrin, alpha 5 (fibronectin receptor, alpha polypeptide)	2.554	0.00151	NM_002205
Protein tyrosine phosphatase, non-receptor type substrate 1	2.38	0.00252	NM_080792
Integrin-linked kinase-associated serine/threonine phosphatase 2C	2.107	0.00141	NM_030768
A disintegrin-like and metalloprotease with thrombospondin type 1 motif, 1	2.081	0.0285	NM_006988
Chemokine (C-X3-C motif) ligand 1	-3.068	0.000683	NM_002996
Thrombospondin 1	-2.883	0.0163	NM_003246
Hyaluronan synthase 1	-2.654	0.00277	NM_001523
Collagen, type VIII, alpha 1	-2.594	0.0103	NM_001850
Plakophilin 4	-2.443	0.0446	NM_003628
Laminin, beta 1	-2.443	0.0023	NM_002291
Discoidin domain receptor family, member 2	-2.306	0.00879	NM_006182
Integrin, beta-like 1 (with EGF-like repeat domains)	-2.137	0.012	AB008375
Cadherin 11, type 2, OB-cadherin (osteoblast)	-2.04	0.00555	NM_033664

Genes involved in cell adhesion that were significantly up- or down-regulated more than two fold after CMS in TM cells. Columns show the gene name, the fold expression change determined by microarray analysis, the calculated p value, where p<0.05 was considered to be statistically significant, and the GeneBank accession number.

The original array data files are available at the Gene Expression Omnibus (GEO) under accession number GSE14768.

### Validation of microarray results by quantitative polymerase chain reaction

Twelve genes upregulated or downregulated by more than twofold expression were further analyzed by quantitative PCR in HTM cells. Although, the precise fold change values observed by Q-PCR were different from those in the gene arrays, the results were in general agreement with the arrays for all the analyzed genes (Table 7). In addition, six upregulated genes (*MMP3*, interleukin 8 [*IL8*], *VEGFC*, *HSP70*, bone morphogenetic protein 2 [*BMP2*], and *ECSM1*) were validated in pTM cells to further analyze the effect of inhibitors on gene expression during mechanical stress.

**Table 7:** Comparison of fold changes between arrays and Q-PCR after CMS in HTM cells.

**Gene**	**Q–PCR fold**	**p value**	**Array fold**	**p value**
*HSP70B'*	200.81	6.35E-05	489.4	0.0206
*ECSM1*	70.92	1.07E-04	33.41	0.000613
*MMP3*	44.67	1.32E-04	29.78	1.13E-05
Hyaluronan Synthase	16.57	4.85E-04	5.38	0.00474
*BMP2*	38.87	7.98E-05	20.07	0.000553
Reg. G protein 20	30.76	3.33E-05	53.81	0.000828
*VEGFC*	4.65	0.00167	5.92	7.05E-05
*MMP10*	255.31	8.90E-05	26.77	0.0138
*NRFFa4a1*	-258.07	5.35E-04	−12.58	0.000121
*EGR3*	-47.5	5.99E-05	−8.31	0.000761
*BTEbp-1*	-52.14	1.18E-04	−13.88	0.0022
JunB protooncogene	-41.66	1.18E-04	−4.67	0.00183

Genes whose expression changes after CMS was validated by Q-PCR. Columns show gene name, fold of expression change after CMS determined by Q-PCR analysis, the p-value for Q-PCR analysis calculated using the ANOVA test for the differences in means between the normalized C_T_ values in stretched cells versus non-stretched controls, fold expression change determined by microarray analysis, and their corresponding p-value.

### Cell viability

After CMS (6 h -15% stretching, one cycle/s), both HTM and pTM cells showed only a small decrease in viability (5% and 7%, respectively) when compared to cells in the same conditions without stress.

### Effect of inhibitors on gene expression during mechanical stress

Chemical inhibitors were used to analyze the effect of the MAP kinase pathways (p38, ERK, JNK),  phosphoinositide 3-kinase (PI3) pathway, PKA pathway, and P2Y receptor signaling pathway on gene expression changes induced by mechanical stress. We evaluated the effects of these inhibitors upon the induction of some ECM regulator/modifier genes (*MMP3*, *BMP2*, and *ECSM1*), the most upregulated stress response gene on the array (*HSP70B’*), one inflammatory response gene (*IL8*), and one growth factor gene (*VEGFC*).

**Figure 1:**
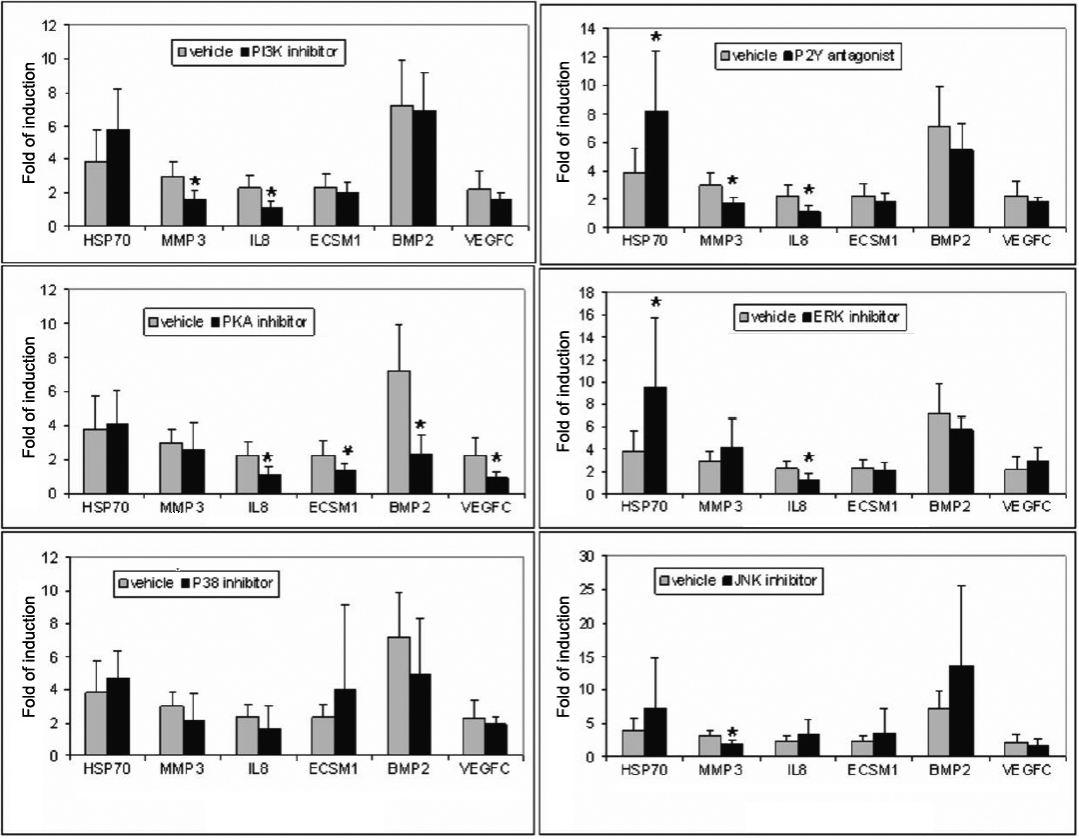
Effects of inhibition of p38, MEK/ERK, JNK, PKA, PI3K, and P2 receptor pathways on the upregulation of six genes induced by CMS. Porcine TM cells were subjected to CMS (15%/1 hrz) for 6 h in the presence or absence of the following inhibitors SB203580 (**A**; p38), PD98059 (**B**; MEK/ERK), SP600125 (**C**; JNK), H89 (**D**; PKA), wortmannin (**E**; PI3K), and suramin (**F**; P2 receptors). The effects of these inhibitors on the upregulation of six genes (*HSP70*, *MMP3*, *IL8*, *ECSM1*, *BMP2*, and *VEGFC*) induced by CMS was evaluated by Q-PCR. Data represents the fold of induction for each gene in the absence or presence of the respective inhibitor (10 µM; n=5). An asterisk indicates that p is less than or equal to 0.05 using a paired Student’s *t* test.

The inhibition of the MAP kinase pathways (p38, JNK, and ERK) resulted in the CMS-mediated induction of the heat shock protein 70 (*HSP70*), but this effect was only statistically significant for the inhibition of ERK. The induction of *IL8* was enhanced with the JNK inhibitor. ERK and JNK inhibitors decreased the level of induction of *IL8* and *MMP3*, respectively. PKA pathway inhibition reduced the induction of *IL8*, *ECSM1*, *BMP2*, and *VEGFC*. Inhibition of the PI3 pathway affected the induction of *MMP3* and *IL8*. The P2Y receptor signaling pathway inhibitor, suramin, also decreased the induction of *MMP3* and *IL8*. Treatment with suramin also resulted in the induction of *HSP70* (Figure 1A-F).

## Discussion

Our results document that CMS induced an intricate response in cultured TM cells with extensive gene expression changes. Some of these changes could participate in outflow regulator mechanisms and could be part of a homeostatic mechanism to maintain normal IOP levels. Others probably serve to maintain cellular integrity from mechanical stress.

Although some changes such as the induction of *MMP3* have been reported in other models of mechanical stress [7], the overall pattern of gene expression associated with cyclic mechanical stress was clearly different from that reported in similar experiments using static stress. One factor that could potentially lead to such differences is the cell type used in the experiments. While Vittal et al. [9] used porcine TM cells, our study was conducted using human TM cells. However, Q-PCR analyses of six genes upregulated in human cells were also upregulated in porcine cells, suggesting that the responses in these two species may be similar. Therefore, it appears more likely that the type of stress and the specific experimental conditions could play a more relevant role in our observed results.

Some of the more prominent changes in gene expression induced by cyclic mechanical stress affected genes involved in cellular protection against different types of stress. In particular, these changes included upregulation of the HSP70 family. This family of HSPs comprises several highly evolutionary conserved proteins with different levels of inducibility in response to metabolic stress that are known to provide cytoprotection to cells, making them resistant to otherwise lethal levels of stress. HSP70 proteins maintain cell survival through the regulation of multiple steps within apoptotic pathways (i.e. stress activated protein kinase [SAPK] and JNK), and they are also believed to regulate key upstream mediators of apoptosis including oxidative stress and Ca^2+^ overload. The HSP70 family member exhibiting higher levels of mRNA induction in our model was *HSP70B’*, which is characterized by tight regulation and high inducibility. This protein is transiently induced in response to stress and then rapidly degraded by the proteasome system [14].

The observed upregulation of genes such as *ECSM1* (endocan) and *VEGFC* could potentially be associated with the mitogenic effects that mechanical stress produces in some other cell types [15]. ECSM1 is a dermatan sulfate proteoglycan that promotes mitogenic activity through interaction with hepatocyte growth factor/scatter during embryonic development and tissue regeneration [16]. The VEGF family of proteins can also exert mitogenic responses and is implicated in embryogenesis and tissue regeneration [17,18]. VEGFC has been specifically determined to be required for the development of the vascular and lymphatic systems [19]. The activation of mitogenic responses by CMS in the TM together with the induction of protective mechanisms (e.g., HSP70) could help to explain the observation that in organ culture, the TM did not show a net cell loss but rather an increase in cellularity after CMS [12].

Cyclic mechanical stress induced changes in a large number of genes that are known to affect the outflow facility such as those influencing the composition of the ECM, cellular cytoskeleton, and cell adhesion. However, some of these changes might be expected to exert contradictory effects in outflow facility. For instance, while the relatively large induction observed in *MMP3* would be expected to increase aqueous humor outflow facility [20], the upregulation of *BMP2* would be more likely to decrease outflow facility [21]. The induction of some MMPs by mechanical stress has been hypothesized to be part of a homeostatic response aimed at increasing outflow facility after an increase in IOP [10]. On the other hand, BMP2 have been shown to increase ECM deposition, and BMP2 activity in HTM cells has been proposed to contribute to outflow resistance by the induction of osteogenic factors during aging and glaucoma [21-23].

In our model, CMS elicits responses that could potentially increase outflow facility together with other responses that are associated with the need to maintain tissue integrity in the presence of mechanical forces. While the first set of responses may help to prevent abnormal elevations of IOP, the second could potentially contribute to increased rigidity of the TM over time and lead to increased outflow resistance. A similar combination of homeostatic and pathogenic effects induced by mechanical stress have been well documented in other tissues including the vascular system [24-26].

To gain insight into the regulatory mechanisms governing the observed responses to CMS, we analyzed the effects of the inhibition of several regulatory pathways on the induction of six relevant genes upregulated after CMS (*HSP70B’*, *VEGFC*, *MMP3*, *BMP2*, *ECSM1*, and *IL8*). Since the activation of inflammatory cytokines has been previously reported as a potentially important factor associated with CMS [13,27,28], we also evaluated the effects of these inhibitors on the expression of *IL-8*, which was the inflammatory cytokine most upregulated in our model.

MAPK and cytokines have previously been reported to be affected by mechanical stress and to induce MMPs in the TM [8,13,29,30]. Our model supports the involvement of ERK in the induction of *IL8* and JNK in the induction of *MMP3*. These two pathways may also have an inhibitory effect in the induction of *HSP70*. However, general MAPK inhibitors do not provide information about specific isoforms of the different MAPKs, which may play different roles. More specific analysis will be necessary to clarify the role of the MAPK isoforms in the responses to CMS in the TM. The role of PI3K/AKT and P2 receptors in the induction of *MMP3* and *IL8* but not in other responses associated with tissue damage and regeneration, (e.g., *ECSM1*, *VEGFC*, and *BMP2*) suggests that PI3K/AKT and P2 receptors could potentially play a contributing role to homeostatic responses aimed at lowering IOP. Protective responses aimed at preventing tissue damage and regeneration such as the induction of *ECSM1* and *BMP2* could potentially be induced by the PKA/cAMP pathway.

All together, our results show that TM cells exposed to CMS manifest extensive changes in gene expression. Some of these changes, such as the upregulation of *MMP3*, have the potential to increase outflow facility and could be part of an homeostatic mechanism involved in the maintenance of normal IOP levels. Other changes are more likely to be related to protective responses aimed at preventing cell and tissue damage (e.g., *HSP70*, *ECSM1*, and *BMP2*). Our results also show that several regulatory pathways may contribute to the diverse responses induced by CMS. The relative contribution of each of these pathways to the gene expression changes induced by mechanical stress may depend on the specific experimental model, which could help explain the variation in results obtained using different models. It is also possible that most in vitro models may not reflect accurately the balance between responses associated with tissue protection and those involved in the modulation of outflow facility.

In conclusion, the effects of CMS on TM cells seem to include a complex set of responses. While some of these responses may contribute to an increase in outflow facility, others that are perhaps aimed at preserving tissue integrity from mechanical damage may have opposite effects on outflow facility. Since the TM is subjected to CMS in vivo, elucidating the mechanisms that protect the cells against mechanical damage and those induced in outflow facility homeostasis may provide important insight into both normal and pathophysiological outflow function.
